# Supplementary material for: Courses of depressive symptoms and diabetes incidence among middle-aged and older adults: A prospective study
Source: PLoS One. 2025 Apr 16;20(4):e0321712. doi: 10.1371/journal.pone.0321712 (PMC12002524; doi:10.1371/journal.pone.0321712)
Supplement: Attachment — Submitted filename: Review.pdf [file pone.0321712.s001.pdf]

Summary of the research and overall impression

Depression can be a risk factor for type 2 diabetes mellitus (DM). The severity and duration of depression can affect to the incidence of the outcome: DM2. In this relative novel and indeed interesting manuscript the authors continue the investigation between the relationship of depressive sympoms and DM. It certainly is a strenght in the current study that the overall data seems to be robust and expecially that there are multiple time points were the individuals depressive symptoms are measured. Multiple measures related to depressive symptoms gave the possibility to seek trends. Follow-up time is not short but it is not markable long neither. Some weaknesses can be pointed out: depressive symptoms are measured biannually so there is a reasonable possibility that all the recurrence in the depressive symptoms is not seen. Another weakness can be consided to be that the severity of the depression is not measured in a diagnostic point of view. Third the study outcome was only based on individuals own memory (not register data, healthcare records made by licenciated physician, prescripted mecidation for DM2 treatment etc.). The study popultion was mainly white and english speaking and is one reason why the results could differ in different populations.

Guidance for the review comments:

1. Order of review comment: line number (e.g. 25) in the manuscript, possible sitation ("Depression...") from the manuscript and reviewers comments &/ questions

2. Terminology: **major** = major issue to consider, minor = minor issue to consider

3. Questions after word *optional* are optional so no comments is an optional answer for those questions

Comments to the Authors

Did the authors consider doing sensitivity analysis with higher than four (4) points cut-off for CES-D? Further how is the cut-off score for the main analysis justified? If the cut-off score for depressive symptoms is lowered is there a higher risk for other reasons behind the incident DM than depressive symptoms?

In the main model remitted depressive symptoms and variable course depressive symptoms were associated with incident diabetes after adjusting for covariates. Yet in the sensitivity analysis the adjusted model indicated that incident depressive symptoms without remission, variable course depressive symptoms and chronic depressive symptoms were associated with incident diabetes. The discrepance between the results is not fully discussed in the manuscript.

At least in the discussion section it should probably be mentioned that variable course depressive symptoms might be related not only for depression but generally to a more severe disease called bipolar depression - which may have different impact to the DM risk (reference).

Ethics statement

Minor: "Carleton University Research Ethics Board-B also approved these analyses (Project # 117637)." Word "also": Who else approved the analyses? Further does the approval of analyses mean approval beforehand (protocol) or afterwards (after conducting the analysis with the original data and having the same results)? "Participants provided informed consent verbally to participate in the interview portion of ELSA..." Why informed consent was not taken in writing?

Optional: How was the identity of each participant confirmed (previously known, passport inspection etc.) and what was the education level and speciality (health care?) of the trained interviewers and fieldworkers? Even further how experienced the interviewers and fieldworkers what comes to same kind of tasks before. What kind of training was provided specifically for the task, interview? How many interviewers and fieldworkers the was? Was there need to replace any workers during the study and if so did the new workers get excatly the same education? Was the work monitored during the study and if so what kind of things or issues the was for discussion? How long is the source data preserved?

Data Availability

Minor: What is the reason for data availability restriction? Hypothetically, could the reviewer have the original data to conduct and scrutinise the analysis for the reproductability valuation?

Optional: What precise prosedures should be followed to get the data for conducting the analysis and how long time it would take before everything would be ready for the reproductability valuation?

Introduction

30, minor: "Approximately 10% of adults worldwide have diabetes (1). Diabetes is more common among middle-aged and older adults relative to other age groups." Consider word "younger" instead the word "other".

34, minor: "Elevated depressive symptoms are a risk factor for diabetes;" Consider words "Depression symptoms" instead "Elevated depressive symptoms"

34-36, **major**: Check references 3 and 26, 27 and correct the discrepancy in the manuscript

45-46, minor: "In particular, the persistence, remittance, and onset of depressive symptoms have been differentially linked to physical health outcomes." Consider to mention the following: There might be multiple reasons why the onset of depression relates to the incidence of DM. Onset can relate to the overall duration of depression as well to the age of an individual during the onset of depression and to the era (different era, e.g. 1980 vs. 2010, might indicate different diagnostic tools and treatment possibilities, perhaps different treatment outcomes etc. as well).

54-56, **major**: "Behavioural...". Also antidepressants may increase weight gain (reference).

56-59, **major**: "Specific...". Longer duration of depression may indicate more severe depression disease and/or less (available?) treatment.

Methods

102-108, minor: Due to the data measurement method/possibility there are gaps between the biannual measurement points. Onsets of the exposure, depression, not noticed/recorded in a real time. So during the every two years blind time (from the authors point of view) there might potentially be both depressive symptoms and recovery from the symptoms in a way that the study exposure is not detected at all. More succint: depression symptoms between biannual measurements remained undetected in the current study.

133, *optional*: "Variable course" might also be called relapsing-remitting.

136-137, **major**: "Participants were asked if they had ever been told by a doctor that they have diabetes or high blood sugar." High blood sugar can occur (shortly) without diabetes, e.g. due to an infection but still considered as a positive outcome? If high sugar is measured by a nurse and the individual is still waiting to see the doctor, when the question is asked, is it still considered a negative outcome?

148-149, minor: "Participants lost to follow-up or who were alive at the end of the follow-up period were censored." Is withdrawal of the consent also considered as lost to follow-up? One could argue that there should not occur interval censoring but right censoring is possible in the current study.

Discussion

214-233, *optional*: this part could possibly be shorter.

222-224, **major**: "increasing depressive symptoms group was at highest risk of diabetes (16). The latter finding is consistent with the present finding from the sensitivity analysis that chronically elevated depressive symptoms were associated with incident diabetes." Can one compare "increasing depressive symptoms" and chronically elevated depressive symptoms"? Severity of depression (graded with less or more symptoms) might not be the same as duration of depressive symptoms.

259-261, **major**: "The two courses of depressive symptoms that were most consistently associated with incident diabetes in the present study (i.e., chronically elevated, variable course)..." But in the main analysis chronic depressive symptoms did not associate with incident diabetes after adjusting for covariates!

279, minor: There is a missing dot in the line.

287, minor: There is a missing space in the line.

284, **major**: Consider replacing the word "some" with the key findings of the current study.

Tables, *optional*: Consider adding space to the first columns of the tables to get even spaces between the rows.
