# Supplementary material for: Courses of depressive symptoms and diabetes incidence among middle-aged and older adults: A prospective study
Source: PLoS One. 2025 Apr 16;20(4):e0321712. doi: 10.1371/journal.pone.0321712 (PMC12002524; doi:10.1371/journal.pone.0321712)
Supplement: Attachment — Submitted filename: PLos 1 Review - Evaluation.docx [file pone.0321712.s002.docx]

**Evaluation & Suggested Improvements:**

**1) Abstract:**

1. Add more context to the background about the courses of depression with time affects the physical health with diabetes among elderly population based on existing studies.

2. Mentioning the study design, the age of the study population and the type of study (for example Community based study) would have a clear idea about the method. While statistical tools are mentioned, it does not mention which Statistical software was used with their version and license.

3. Include more specific figures or effect sizes (in terms of percentage or fold increases) in the results. To be more complete, the Specific courses of depressive symptoms be mentioned with their ICD-10/DSM-5 codes.

4. Strengthen the conclusion by recommending targeted interventions based on the study findings.

**Overall, both the title and abstract are clear but could be refined to be more precise, academic, and impactful.**

**2) Introduction:**

1. To improve comprehension from a border viewpoint, emphasize on the operational definitions for depressive symptoms as research-based framework, ideally aligned with the DSM-5 or ICD-10 while incorporating specific terms like "variable course depressive symptoms" and "remitted course depressive symptoms". Similarly Incident diabetes or Type 2 diabetes mellitus has to be fixed throughout the study and defined scientifically with their standard diagnostic procedures established by the WHO guidelines.

**3) Methods:**

1. What sample strategy was employed while face to face interview? Were the paper-based questionnaires well-structured and validated by experts to preserve the study's validity and reliability over the course of the ten-year follow-up involving middle-aged and older persons.

2. Along with the mental and physical health status, were the other anthropometric measurements including systolic and diastolic blood pressure, BMI and waist and hip size recorded during the follow up session? As these parameters shows different patterns with age which affects the overall health status including incident diabetes among the elders.

3. To uphold scientific standards, define incident diabetes in accordance with the American Diabetes Association's recommendations or the WHO standard procedure, coordinating their codes with the DSM-5 or ICD-10 and the methodology followed during the participant's diagnosis.

4. Where the research ethics approvals and written informed consent from participants renewed at every follow-up session?

5. Mentioning the inclusive and exclusive criteria would bring more clarity into the study methodology.

6. For better understanding the method used for selection process can be presented with a flowchart.

7. On what basis was the study sample size determined? Was any pilot study carried out while diagnosing for the incident diabetes?

8. Under Measures, the 8-item on which the modified version of the Centre for Epidemiologic Studies-Depression Scale (CES-D) is based can be expressed in detail as these behaviours/emotions focuses on core depressive symptoms such as mood, sleep, appetite, and motivation, typically retaining items that best represent the underlying construct.

9. Under covariates, listing out all the potential confronters and potential mediators separately could give a better understanding of the analysis.

10. Why only the socio-demographic variables and not the physical parameters measured (as per the database) at baseline considered and adjusted for confronting factors while running the Cox proportional hazard ratio model?

11. Statistical analysis are carried out systematically and carefully, bearing the main goal of the study. Mention the software or the application used to carry out the statistical analysis with their version and license.

12. Mentioning Cronbach's alpha assesses the internal consistency reliability of the CES-D scale which effectively calculates the scale’s items measure the same underlying construct-depressive symptoms.

**4)Results:**

Health is defined as a state of complete physical, mental, and social well-being (WHO,1998). Bearing this in mind and considering the broad aims and scopes of the journal – PLOS ONE, it is not fair to consider only one aspect of health.

1.Was the study impeded by the effects of antidepressant medications? If so, how was it handled?

2. Since older age groups are predisposed to neuropsychiatric illnesses, is it possible to draw a line where we can say with conviction that the symptoms of, say, depression are due to diabetes only and not to old age-related other complications?

3. The presence of diabetic complications (BP levels, obesity, anaemia, etc.) or family history does not seem to be associated with depression, why?

4. The results are presented mostly in text form, but there is limited use of graphs or figures to visually represent key findings. For example, presenting a graph comparing different courses of depressive symptoms along the timeline, would make the longitudinal data more engaging and easier to interpret at a glance.

**5) Discussion and Conclusion**:

1. When the study discusses that “…clinically meaningful courses…” then defining the specific courses of depressive symptoms and incident diabetes based on established diagnostic guidelines (e.g., DSM-5, ICD-10) becomes critical as to ensures consistency in identifying and measuring these conditions across participants.

2. Despite reports of statistical significance (e.g. HR > 50%), the clinical relevance remains underexplored. For instance, while variable depressive symptoms are linked to incident diabetes, the implications for patient care and treatment are unclear. A deeper discussion on the real-world impact is needed.

3. Indeed, it was worth noting to performed sensitivity analyses, as deviating from any standard protocol needs clear justification. Then the same applies when confronting with the potential physical parameters including the anti-depressive drug effect or the family history or the other complications associated with the age of the participants. Justify?

3. The link between depression symptoms and diabetes may be driven by continuous exposure to underlying physiological and/or behavioral mechanisms.” – agreed, but previous research has shown a reciprocal association between diabetes and depression. Justify?

4. Instead of only quoting the references and naming the mechanisms behind the association between diabetes and the trajectories of depressive symptoms, if the association is demonstrated with possible causes such as vascular alterations or hormonal variations in older persons with diabetes would be much recommended in the discussion section as it could strengthen the study's interpretation and depth.

5. Future studies is recommended with directions that could be more specific, focusing on key gaps in the study.
